# Finding food in a changing world: Small‐scale foraging habitat preferences of an insectivorous passerine in the Alps

**DOI:** 10.1002/ece3.10084

**Published:** 2023-05-17

**Authors:** Thomas M. Müller, Christoph M. Meier, Florian Knaus, Pius Korner, Barbara Helm, Valentin Amrhein, Yann Rime

**Affiliations:** ^1^ Swiss Ornithological Institute Sempach Switzerland; ^2^ Department of Environmental Systems Sciences Swiss Federal Institute of Technology Zurich (ETH Zurich) Zurich Switzerland; ^3^ Department of Environmental Sciences, Zoology University of Basel Basel Switzerland

**Keywords:** alpine birds, elevation, ground cover, habitat heterogeneity, insectivorous

## Abstract

Organisms living in high‐elevation habitats are usually habitat specialists who occupy a narrow ecological niche. To envision the response of alpine species to a changing environment, it is fundamental to understand their habitat preferences on multiple spatial and temporal scales. However, information on small‐scale habitat use is still widely lacking. We investigated the foraging habitat preferences of the migratory northern wheatear *Oenanthe oenanthe* during the entire presence at a breeding site in the central Alps. We repeatedly observed 121 adult and juvenile individuals. We applied Bayesian logistic regression models to investigate which habitat characteristics influenced foraging habitat selection on a fine spatial scale, and how habitat use varied temporally. Throughout their presence on the breeding grounds, northern wheatears showed a consistent preference for a mosaic of stones and bare ground patches with slow‐growing, short vegetation. The proximity of marmot burrows was preferred, whereas dense and low woody vegetation was avoided. After arrival at the breeding site, short vegetation, preferably close to the snow, was favored. The preference for open habitat patches that provide access to prey underlines the critical role of small‐scale habitat heterogeneity for northern wheatears. The strong and consistent preference for a habitat that is under pressure from land‐use and climate change suggests that this alpine bird species may be sensitive to habitat loss, leading to a potential range contraction. We highlight the need to conserve habitat diversity on a small spatial scale to ensure the long‐term availability of suitable habitat for northern wheatears in the Alps.

## Graphical abstract

Repeated observations of northern wheatears (*Oenanthe oenanthe*) in their Alpine breeding range underline the importance of small‐scale habitat heterogeneity on the availability of suitable foraging habitat for this alpine passerine. The study revealed that a mosaic of short vegetation and bare ground patches within more productive areas are favored throughout the presence of the species at the breeding site. Conservation of structural heterogeneity that is affected by climate and land‐use change is crucial, as Alpine areas are becoming increasingly important for this habitat specialist.

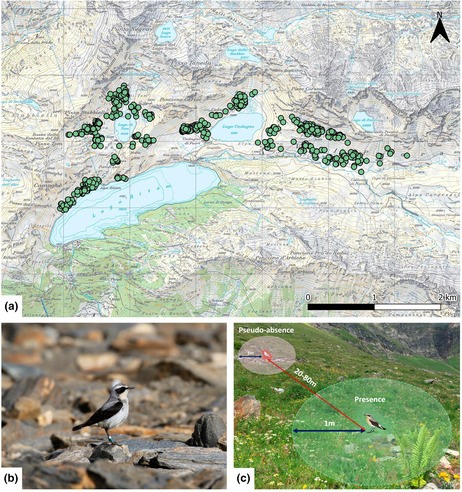

## 1 INTRODUCTION

The ecological niche of a species is defined on multiple spatial and temporal scales (Mahon et al., 2016). Hence, to understand or preserve a species, it is necessary to identify its relevant habitat preferences from large‐scale distributions to small‐scale habitat features. The availability of suitable foraging habitat plays a special role in the niche configuration and is crucial for survival and successful reproduction. More specifically, food availability, comprised of food abundance and accessibility, is a major driver of foraging habitat selection that is influenced by habitat features on a fine scale (Arlettaz, 1999; Barras et al., 2020; Cody, 1985; Dussault et al., 2005). Food abundance and accessibility, however, are often promoted by different habitat characteristics and are temporally variable (Atkinson et al., 2004; Dussault et al., 2005; Fuller et al., 2007). Particularly, species with narrow requirements, so‐called specialists, are expected to be relatively sensitive to changes in food availability (McPeek, 1996). Typically, alpine species are often adapted to a short vegetation period, and they are restricted to a higher elevational range that is characterized by habitat heterogeneity on a finer scale, compared to lowland habitats (Cortés & Wheeler, 2018). For insectivorous alpine birds, prey abundance is driven by a stronger seasonality at high elevation (Pilar et al., 2020; Resano‐Mayor et al., 2019). Arthropod abundance, diversity, and species richness peak in early summer and then decrease to relatively low levels until autumn (Pilar et al., 2020). Consequently, the time window is limited for prey availability to match food demand for brood provisioning, for expensive maintenance such as molt, and for juvenile post‐fledging establishment (Arlt & Pärt, 2008; Resano‐Mayor et al., 2019; Tulp & Schekkerman, 2008).

Alpine regions are more vulnerable to climate change than low‐elevation areas (Brunetti et al., 2009). They experience adverse effects of rising temperatures, altered precipitation patterns, as well as advanced snowmelt and vegetation development that lead to an upward shift of the treeline (Gehrig‐Fasel et al., 2007; Gobiet et al., 2014; Keller et al., 2005; Theurillat & Guisan, 2001). Moreover, land‐use changes influence vegetation development in alpine areas through two opposed processes (Kulakowski et al., 2011): Low‐intensity agricultural activities such as livestock grazing are being abandoned, leading to bush encroachment and ultimately to forest encroachment (Baur et al., 2006), while areas that are still managed tend to undergo agricultural intensification (Fischer et al., 2008). Land‐use and climate change have fundamental effects on the majority of organisms across trophic levels, through either the loss of suitable habitat or shifting vegetation phenology (Ferrarini et al., 2017; Hughes, 2000; Inouye, 2020; Keller et al., 2005). For migratory birds in particular, advanced vegetation phenology can lead to a potential phenological mismatch (Jones & Cresswell, 2010; Saino et al., 2011; Visser et al., 2004), because it reduces prey accessibility for ground‐foraging species as a result of increased vegetation height and of advances in the peak in arthropod abundance (Renner & Zohner, 2018; Tulp & Schekkerman, 2008).

As a long‐distance migratory songbird, the northern wheatear (*Oenanthe oenanthe*) is affected by changing habitat characteristics and shifting vegetation and prey phenology on multiple spatial and temporal scales (Jähnig et al., 2020; Sander et al., 2021, 2022). The species has a circumpolar distribution and overwinters in sub‐Saharan Africa (Bairlein et al., 2012; Dunn et al., 2020; Meier et al., 2022; Rime et al., 2023). In Northern European lowland breeding sites, where seasonality is less strong compared to alpine habitats, northern wheatears favor open fields with short vegetation (Arlt et al., 2008; Arlt & Pärt, 2007; Paquet et al., 2019) and seem to be more limited by prey accessibility than by prey abundance (van Oosten et al., 2014). Unlike lowland breeding ranges, in Switzerland, the species is limited to high elevations above the tree line (Knaus et al., 2018). While in most parts of Europe, northern wheatear populations are declining, the Swiss Alpine population is stable overall while experiencing an upward shift in the elevational distribution (Hallman et al., 2022; Keller et al., 2020; Knaus et al., 2018). The population trend of the Alpine northern wheatear population points toward an increasingly important role of alpine habitats for the conservation of this species in central Europe (Knaus et al., 2018). This Alpine population faces spatial and temporal landscape dynamics that are different from those in the European lowland (Brunetti et al., 2009; Pilar et al., 2020). To examine the sensitivity of the species to current and future habitat changes and shifting vegetation phenology in the Alps, it is important to understand how the species interacts with the highly seasonal and variable habitat that the alpine ecosystem provides on a fine spatial and temporal scale.

Here, we conducted an observational study on uniquely identifiable individuals to determine the preferred foraging habitat of northern wheatears in their Alpine breeding range throughout their stay. We focused on the microhabitat at foraging locations and compared it with the available habitat at random locations within the territory. We investigated the role of vegetation height and ground cover composition in providing accessibility to prey. Foraging preferences may change throughout the annual cycle. Therefore, we considered the birds' entire presence at the breeding site, including during the pre‐breeding and postbreeding periods. This also covers key processes such as molt and premigratory fuel deposition, as well as the high‐risk phase of post‐fledging establishment of juveniles. To determine the role of prey accessibility on Alpine breeding grounds, we examined the importance of small‐scale heterogeneity in providing suitable foraging habitat. Furthermore, we explored the role of grazing cattle and alpine marmots (*Marmota marmota*) in shaping habitat heterogeneity on a small scale.

## 2 METHODS

### 2.1 Study area

**FIGURE 1:**
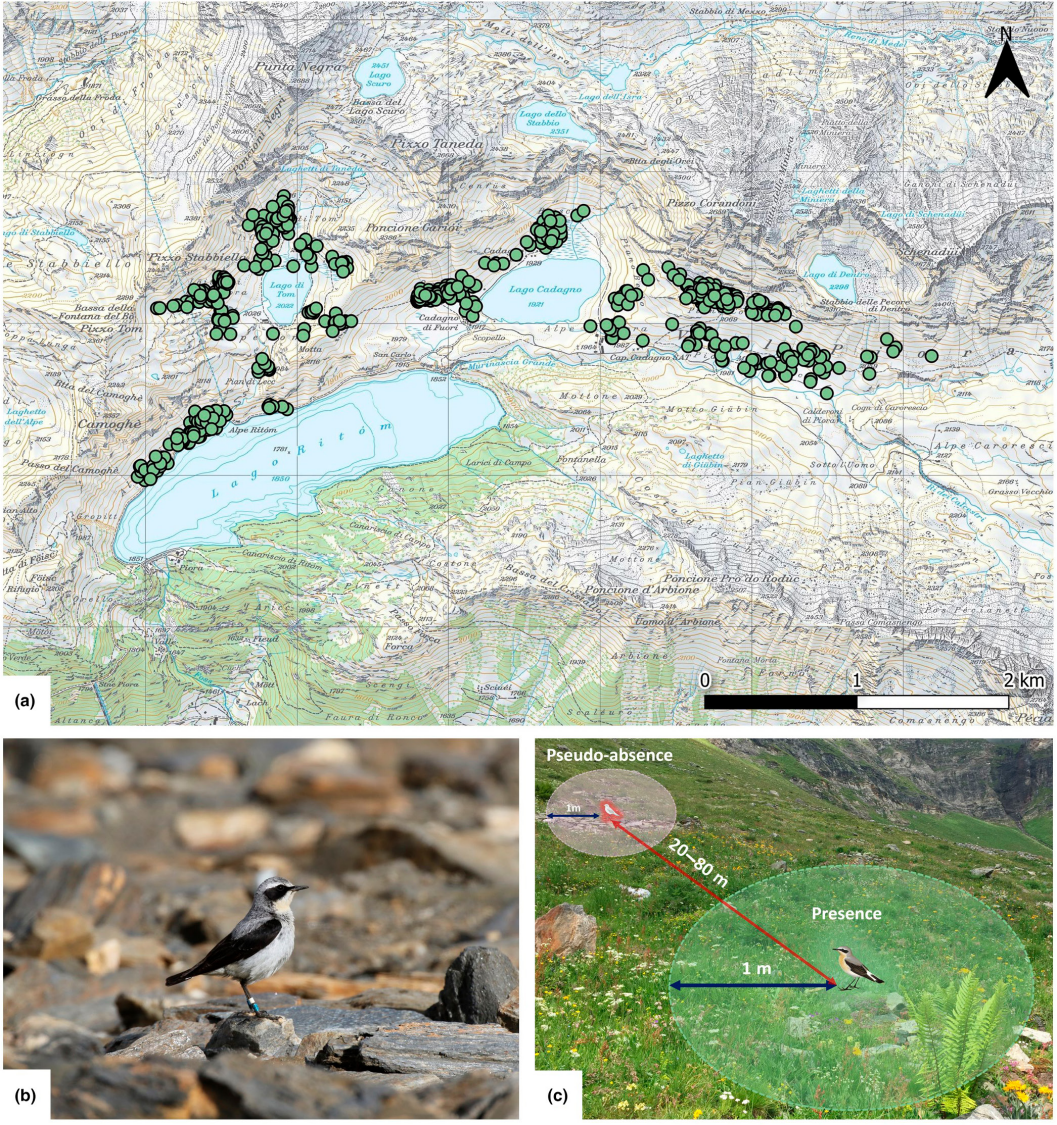
The map (a) shows the position of all foraging points (green dots) recorded in the study area in Val Piora. Foraging habitat data were recorded for color‐ringed northern wheatears (b) on a 1‐m radius around foraging (presence) and pseudo‐absence locations (c). Pseudo‐absence locations were located randomly within 20–80 m and at a random angle (relative to true North) of each foraging location. background map: ©swisstopo, photos: ©Y. Rime.

Our study area is located in Val Piora in the central Swiss Alps (46°33′N 8°42′E, Figure 1). It covers 6 km^2^ of mostly south‐exposed slopes above the tree line, ranging from 1850 to 2200 m.a.s.l. and hosting more than 100 breeding pairs of northern wheatears. The habitat is characterized by heterogenous open grassland interspersed with rocks, boulders, debris fields, and remains of man‐made rockpiles and stonewalls. Between July and September, the pastures are grazed in a rotational manner and the cattle are frequently moved, constituting a low‐intensity grazing regime. The area is usually covered by snow between November and May.

### 2.2 Study design

In the frame of a project on migration and ecology of northern wheatears, individuals have been ringed in the study area since 2010 (Meier et al., 2022; Rime et al., 2023; Schmaljohann et al., 2016). Each bird was ringed with a unique combination of one metal ring and three plastic color rings (Figure 1). Adults were caught using baited spring traps and cage‐traps that were placed at the nest entrance. Where they could be reached, chicks were ringed at their nest once they were 7 days old.

We observed ringed individuals between May 12 and September 03, 2021, covering the arrival, incubation, feeding (period of food provisioning for chicks), and postbreeding stages. Northern wheatears arrive on their Alpine breeding grounds between the end of April and mid‐May and depart for fall migration around mid‐September (Glutz Von Blotzheim & Bauer, 2001; Meier et al., 2022; Rime et al., 2023; Sander et al., 2021). During this period, ringed birds were followed weekly from the distance, using binoculars and a scope, until the first foraging attempt of each observation. We recorded the exact location of the foraging event on a photograph taken through the scope. After the bird had left the foraging location, we immediately mapped the microhabitat on site and recorded the exact coordinates and information on the individual (color ring combination, sex, age, and nest ID) in QField (QGIS Development Team, 2020). To compare the foraging (presence) locations with locations that have not been chosen by the bird, we mapped the microhabitat at a nearby location within a randomly selected distance of 20–80 m to the foraging location at a random angle (0°–360°) for each foraging event (Figure 1, Barbet‐Massin et al., 2012; Johnson, 1980). This distance range was selected to ensure that pseudo‐absence locations were located within the territory of the observed bird (Glutz Von Blotzheim & Bauer, 2001). Adult birds usually remained within their territory throughout their entire stay in the study area, including for foraging activities (Rime et al., 2023). To make sure that each presence‐pseudo‐absence pair is independent, we moved on to the next territory after having recorded all ringed individuals sighted within their territory.

We recorded the following set of habitat variables (Table 1 and Table S1) on a 1‐m radius around foraging (presence) and pseudo‐absence locations (Figure 1): ground cover estimates (percentage of live vegetation, dead vegetation, woody vegetation, bare ground, stones [granulometry >4 mm], and snow) and vegetation height. We calculated the vegetation height using the mean of three representative measurements within the 1‐m radius. Additionally, we estimated the distance to the closest marmot burrow and recorded cattle grazing activity, immediate cattle presence, and presence of cow dung within the 1‐m radius. For each foraging and pseudo‐absence location, we also computed the distance to the nest if it was found, and the normalized difference vegetation index (NDVI) and its rate of change between months. The distance to the nest was calculated based on the SwissALTI3D digital elevation model (swisstopo, 2018) in QGIS (QGIS Development Team, 2020). NDVI raster images for the study area were generated on Google Earth Engine (Gorelick et al., 2017) based on Sentinel‐2 satellite images with a spatial resolution of 10 m (ESA, 2015). After applying a cloud filter (<50% cloud area), the image with the clearest conditions for each month (April–September 2021) was manually selected, and the NDVI values were extracted in R (R Core Team, 2021) using the *extract* function from the package *raster* (Hijmans, 2021). To detect local shifts in greenness, the rate of NDVI change was computed as the difference between the NDVI values extracted from the images of the previous and the following month of the foraging event at each foraging and pseudo‐absence location. To allow for a comparison between different habitat scales, we additionally recorded the same set of variables on a 2‐m radius around the foraging and pseudo‐absence locations.

**TABLE 1:** Predictors used to model the foraging probability (presence vs. pseudo‐absence).

Category	Variables	Description	Unit	Analysis				Transformation
				General model (*n* = 542)	Arrival and incubation (*n* = 193)	Feeding (*n* = 190)	Postbreeding (*n* = 182)	
Fixed effects				Mean (range) (2.5%; 97.5%)	Mean (range) (2.5%; 97.5%)	Mean (range) (2.5%; 97.5%)	Mean (range) (2.5%; 97.5%)	
Ground cover	Dead vegetation	Brown vegetation grown in the previous year	%	10 (0–100) (0; 64.9)	25.9 (0–100) (0; 80)	5.3 (0–85) (0; 38.6)	3.6 (0–40) (0; 29.6)	Poly^a^, z^b^
	Woody vegetation	Shrubs and other plants with a woody stem	%	6.2 (0–100) (0; 70)	3.9 (0–93) (0; 40.8)	5.5 (0–100) (0; 67.6)	6.7 (0–100) (0; 76.5)	Poly^a^, z^b^
	Bare ground	Bare ground with no vegetation cover	%	17.3 (0–100) (0; 64.9)	20.9 (0–90) (0; 65)	17.7 (0–100) (0; 70)	12.7 (0–85) (0; 45.9)	Poly^a^, z^b^
	Stones	Stones and rocks (granulometry >4 mm)	%	12.1 (0–100) (0; 65)	9.6 (0–100) (0; 56.1)	10.2 (0–73) (0; 50)	14.3 (0–100) (0; 70)	Poly^a^, z^b^
	Snow cover	Area covered by snow	%	Not included	15.5 (0:100) (0; 100)	Not included	Not included	Poly^a^, z^b^
	Vegetation height	Mean height of three representative measurements	cm	13.24 (0–94) (1; 36)	5.45 (0–55) (0; 18)	14.91 (0–94) (3; 36)	15.58 (0–55) (3; 38)	z^b^
Ecosystem engineers	Distance to marmot burrow	Distance to the closest marmot burrow (if within 100 m)	m	7.93 (0–130) (0; 27)	8.75 (0–60) (0.63; 32.75)	8.88 (0–130) (0.48; 33.05)	6.86 (0–76) (1; 22.85)	z^b^
Vegetation Index	NDVI	Normalized difference vegetation index	Index	0.65 (0–0.92) (0.1; 0.87)	0.38 (0–0.88) (0; 0.81)	0.68 (0–0.92) (0.15; 0.88)	0.72 (0.28–0.9) (0.52; 0.87)	Poly^a^, z^b^
	Rate of NDVI change	Change in vegetation greenness	Index change rate	0.18 (−0.36 to 0.83) (−0.22; 0.69)	0.4 (−0.05 to 0.86) (0; 0.74)	0.2 (−0.32 to 0.82) (−0.08; 0.73)	−0.02 (−0.36 to 0.58) (−0.27; 0.3)	Poly^a^, z^b^

Random effects				No. of levels	No. of levels	No. of levels	AnalysisNo. of levels	
Point info	Point ID	Unique identifier for each point pair		542	193	190	182	
Bird Info	Bird ID	Color ring combination		119	57	72	95	

*Note*: Snow cover was only included in the arrival and incubation model. For each model and variable, the mean per 1‐m radius plot, range, 2.5% quantile, and 97.5% quantile are given, and the applied data transformation is provided. Point ID and bird ID were included as random effects for which the number of levels is given for each model.

^a^ The first two orthogonal polynomials were included.

^b^ Centered to 0 and scaled to 1 SD.

As the birds' needs are expected to change during their presence at the study site, we assigned three stages to each of the foraging events on a per‐breeding pair basis. The arrival and incubation stage lasts until the chicks hatch after an incubation period of 13–15 days (Moreno, 1989a). This is followed by a feeding period that includes feeding chicks 13–15 days in the nest and feeding fledglings for 10 days out of the nest until they become largely independent (Glutz Von Blotzheim & Bauer, 2001; Moreno, 1984). The postbreeding period includes the remaining time until both adults and juveniles depart for fall migration (Arlt & Pärt, 2008). During this period, young wheatears must establish themselves, and both the adults and immatures undergo complete molt and deposit fuel for their long‐distance migratory journey (Arlt & Pärt, 2008; Glutz Von Blotzheim & Bauer, 2001).

In total, we recorded 620 foraging locations and an equal number of pseudo‐absence locations (*n*_tot_ = 1240) during the period of presence of northern wheatears in the study area (Figure 1). We followed 121 ringed individuals (53 adult males, 47 adult females, and 21 juveniles). Sixty‐nine adults were returning individuals ringed in previous years, while 31 adults and 21 juveniles were newly ringed during the study period. We collected data for 193 foraging locations during the arrival and incubation stage, 193 during the feeding stage, and 182 during the postbreeding stage, of which 38 were from juveniles.

### 2.3 Statistical analysis

We modeled the foraging habitat selection by comparing the recorded variables between foraging (presence) and pseudo‐absence locations using logistic regression models (logit‐link function) with presence/absence as a binary outcome variable. In all models, the ground cover estimates, vegetation height, distance to marmot burrow, NDVI, and its rate of change were included as fixed effects. To account for individual preferences and repeated observations of the same individual, we included the bird ID (color ring combination) and the point ID (unique number for each presence/pseudo‐absence pair) as random effects (Korner‐Nievergelt et al., 2015; Laird & Ware, 1982). All statistical analyses were conducted in R (R Core Team, 2021). Models were fitted in a Bayesian framework (Gelman et al., 2013; McElreath, 2016), using the *brm* function from the *brms* package (Bürkner, 2017). For each model, we ran four chains, each with 2000 iterations of which the first 1000 were discarded as the burn‐in period (McElreath, 2016). A prior sensitivity analysis (Figure S1) suggested that the model results were sufficiently robust to changing prior specification (Depaoli & van de Schoot, 2017; Link et al., 2002; Nicenboim et al., 2021). Hence, we chose uninformative priors for our models (Berger, 2006; Kass & Wasserman, 1996; Zhou et al., 2014). For the intercept and the group‐level variances (bird ID and foraging ID), we chose default student‐t priors ($\beta \sim \text{Student}\left(3,0,2.5\right)$) and determined a normal prior distribution for the population‐level effects ($\beta \sim \text{Normal}\left(0,100\right)$).

Prior to modeling, numeric variables were z‐transformed (mean = 0, SD = 1). As we expected nonlinear relationships, we included the first two orthogonal polynomials of the ground cover variables and the vegetation index variables in the models using the *poly* function. We checked for collinearity between covariates by calculating the Spearman's correlation coefficient and did not detect strong collinearity among explanatory variables (all |*r*_*s*_| < .7).

Observations in the field suggested potential differences in foraging habitat preferences between adult and juvenile birds. To detect differential preferences of northern wheatears that are related to their age class (adult, juvenile) or sex (female, male), we applied principal component analysis (PCA) using the variables summarized in Table 1. PCA were generated with the *ggbiplot* R package (Vu, 2011) but did not reveal relevant differences between age classes or sexes (Figure S2). As a result, age and sex class were not included in the models.

To detect stage‐dependent differences in foraging habitat preferences during the study period, we analyzed each of the three stages in a separate model, in addition to a general model including the data from the entire study period. To compare foraging habitat preferences across different scales, we also fitted each of the four models with the data collected on the 2‐m radius around the foraging and pseudo‐absence locations.

Due to the strong seasonality in the study area, snow can only be expected at the beginning of the season. As a result, we only used snow cover in the arrival and incubation model. Furthermore, snow cover may lead to biased relative estimates for the other ground covers. Therefore, all locations containing snow (*n* = 156) were removed from the general model. Whenever foraging locations had to be removed, the corresponding pseudo‐absence location was discarded as well. Because ground cover variables always added up to 100%, they could not all be included in the models. Therefore, the main ground cover component, live vegetation, was not used in the models. Visual data exploration did not suggest differences in the topographic variables between foraging and pseudo‐absence locations, which can be explained by the small distance between them (Figure S3). Therefore, topographic variables were not included in statistical models. Similarly, grazing variables were discarded, as they always fell into the same category due to the small distance between foraging and corresponding pseudo‐absence locations. We did not apply any further model selection steps, and no interactions were considered.

We verified model convergence based on Gelman–Rubin convergence diagnostics and visually confirmed convergence using “trace” plots (MCMC plots; Depaoli & van de Schoot, 2017; Rizzo, 2008). We checked for autocorrelation within the MCMC chains using the *mcmc_plot* function from the *bayesplot* package (Gabry & Mahr, 2021). Additionally, we checked for spatial autocorrelation using bubble plots and semivariograms from the *gstat* package (Gräler et al., 2016). In addition, we calculated the area under the curve (AUC) and visually evaluated the goodness of fit (Figure S4) by comparing the fitted values with the data (Korner‐Nievergelt et al., 2015). For each model, we calculated the conditional and marginal Nakagawa's *R* ^2^ (Nakagawa et al., 2017; Nakagawa & Schielzeth, 2013) using the *performance* package (Lüdecke et al., 2021).

To quantify the effect of each predictor on the foraging probability (probability of presence), we present effect plots for each predictor (Korner‐Nievergelt et al., 2015). To do so, for each draw from the posterior distribution, we calculated the regression line over the range of the variable that is shown in the effect plot. From these regression lines, we used the median as a point estimate regression line and the 2.5% and 97.5% quantiles as 95% credible interval (CrI; Korner‐Nievergelt et al., 2015). When showing the effect of a ground cover variable across its range, the remaining area was divided among the other ground cover variables (including live vegetation) proportional to their mean proportions across all locations (and snow cover was set to zero). This was done due to the unit‐sum constraint of ground cover variables. Data, code, and supplementary material used in this study are available under the DOI: 10.5281 at https://doi.org/10.5281/zenodo.7805040 (Müller et al., 2023).

## 3 RESULTS

Our models revealed a positive effect of short vegetation and bare ground on the foraging probability (presence vs. pseudo‐absence) of northern wheatears after the snow has melted, while the habitat characteristics changed as the season advanced. The most common ground cover type at foraging and pseudo‐absence locations was live vegetation with a mean ± SD of 54.3% ± 30.0%, followed by bare ground (17.3% ± 17.0%), stones (12.1% ± 17.8%), dead vegetation (10.0% ± 17.3%), and woody vegetation (6.2% ± 16.9; Table 1). Snow was only present during the arrival and incubation period (15.5% ± 33.6%; Table 1). Characteristic seasonal developments were observed with decreasing snow, bare ground, and dead vegetation covers, while live vegetation increased as the season advanced (Figure S5).

The birds' foraging and pseudo‐absence locations had a similar average vegetation cover in May and June. However, pseudo‐absence locations rose to a higher level of live vegetation before stabilizing at the beginning of June. After that, mean cover of live vegetation remained higher at pseudo‐absence locations compared to foraging locations until the end of the study period. Nevertheless, the general seasonal patterns followed the same trend in foraging and pseudo‐absence locations (Figure S5). Overall, vegetation height at foraging and pseudo‐absence points had a mean ± SD of 13.24 cm ± 9.46 cm and increased throughout the study period. In accordance with the changing ground cover composition and vegetation development, the mean NDVI value was 0.65 ± 0.19 and increased throughout the season. The mean rate of NDVI change was 0.18 ± 0.24, indicating an increase in vegetation greenness from May to July until it started to decline in August (Figure S5).

Based on AUC values as well as marginal and conditional *R* ^2^, the all‐season model (AUC 0.87, *R* ^2^ marginal .59, *R* ^2^ conditional .6), the arrival and incubation model (AUC 0.83, *R* ^2^ marginal .78, *R* ^2^ conditional .78), the feeding model (food provisioning for chicks, AUC 0.92, *R* ^2^ marginal .88, *R* ^2^ conditional .89), and the postbreeding model (AUC 0.92, *R* ^2^ marginal .74, *R* ^2^ conditional .74), all performed well. The difference between the marginal and the conditional *R* ^2^ was consistently small, indicating a small effect of the random factors (i.e., individual and local preferences).

**FIGURE 2:**
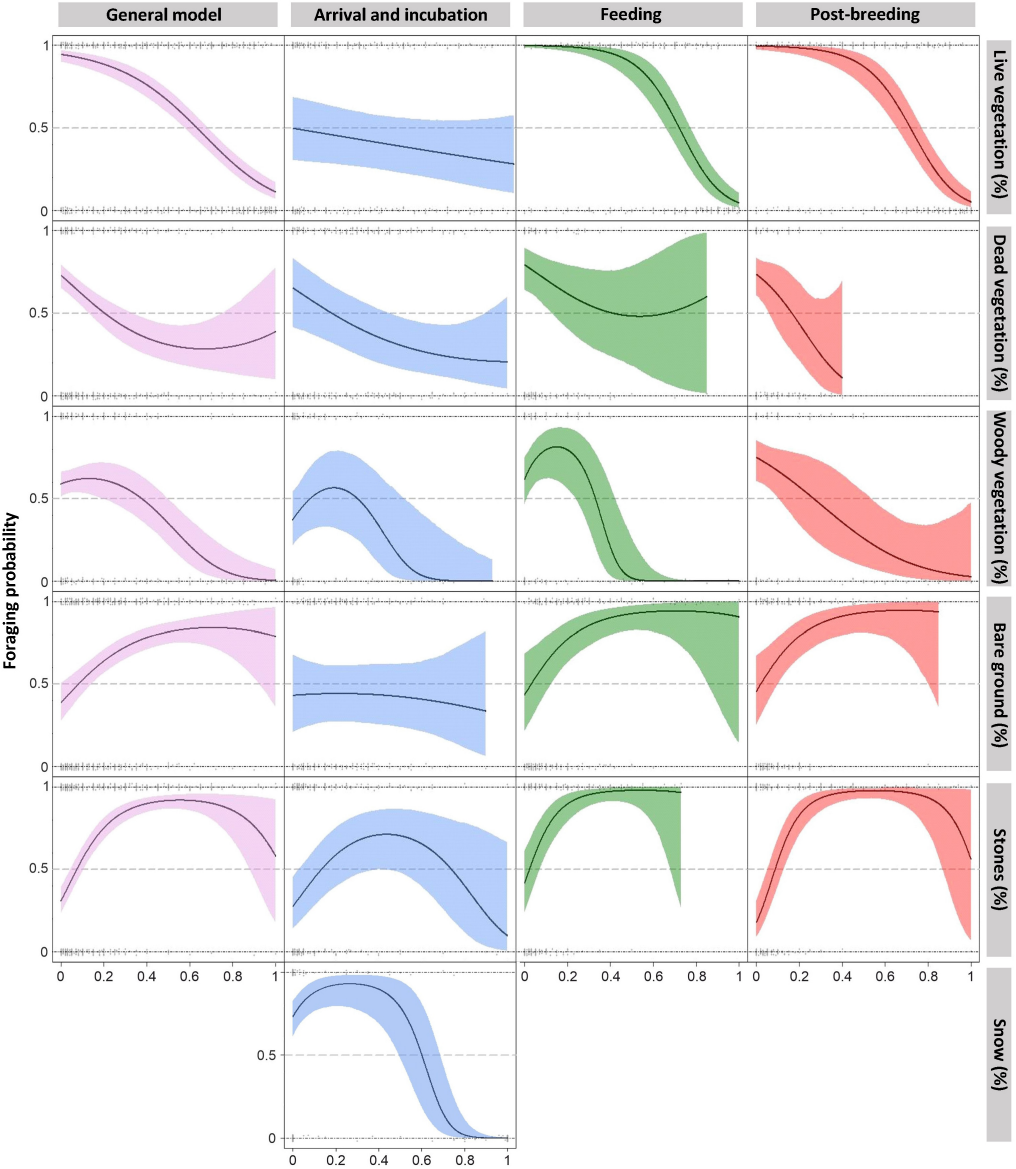
Predictions from logistic regression models showing the average effect (solid line) of each ground cover variable (labeled on the righthand side of the plots) on the foraging probability (presence vs. pseudoabsence) for the general model (whole study period; first column) and each period separately (other columns) within 1 m of the foraging (presence = 1) and pseudo‐absence (0) locations. “Live vegetation” was not used as a predictor in the model but it is a derived parameter from the other ground cover parameters and is given here because all ground covers add up to 100%. The colored areas represent the 95% Bayesian credible intervals and the gray dots show the raw data.

Vegetation height had a strong negative linear effect on the foraging probability (Figure 2), with the effect being strongest while feeding (Table 2). During this period, the mean vegetation height at foraging points was 10.3 cm (±7.3 cm) and 16.2 cm (±10.4 cm) at pseudo‐absence points. Bare ground was positively related to the foraging probability, especially during the feeding period (Table 2, Figure 2). However, no effect of bare ground was found for the arrival and incubation stage when short vegetation and melting snow patches prevailed. Stone cover had a positive effect: during the feeding period, only the linear effect was well supported by the data, whereas in all other models, a maximum probability of foraging was observed at an intermediate (20%–70%) stone cover (Table 2, Figure 2). Locations with low stone cover (<15%) were less likely to be chosen for foraging (Figure 2). In the general model, woody vegetation showed a maximum at a low woody vegetation cover (Figure 2). Especially while feeding, woody vegetation had a negative effect on the foraging probability (Table 2). Locations with more than 40% woody vegetation were never used as foraging locations in the feeding period (Figure 2). In the postbreeding period, woody vegetation only had a weak negative effect (Table 2). Dead vegetation did not play an important role during any of the periods and only showed a weak negative trend in the general as well as the arrival and incubation models (Table 2, Figure 2). During the arrival and incubation period, snow cover showed a strong quadratic effect, indicating a high foraging probability at low to intermediate snow cover levels (Table 2, Figure 2). Locations with more than 60% snow were avoided (Figure 2). Foraging attempts were never observed directly on snow, even when it still covered a large part of the study area.

**TABLE 2:** Summary of the output of the general (all‐season) model, arrival and incubation, feeding (food provisioning for chicks), and postbreeding model using the 1‐m data.

Variables	General model			Arrival and incubation			Feeding			Postbreeding		
	Estimate	CrI		Estimate	CrI		Estimate	CrI		Estimate	CrI	
		2.5%	97.5%		2.5%	97.5%		2.5%	97.5%		2.5%	97.5%
Bird ID (Intercept)	**0.13**	**0.01**	**0.35**	**0.19**	**0.01**	**0.54**	**0.35**	**0.02**	**0.94**	**0.22**	**0.01**	**0.64**
Point ID (Intercept)	0.09	0.00	0.25	**0.14**	**0.01**	**0.41**	**0.22**	**0.01**	**0.64**	**0.21**	**0.01**	**0.62**
Dead vegetation	−0.04	−0.24	0.15	−0.25	−0.64	0.13	0.14	−0.20	0.46	−0.24	−0.62	0.10
Dead vegetation^2^	**0.21**	**0.06**	**0.37**	0.14	−0.15	0.43	0.16	−0.16	0.45	−0.05	−0.39	0.27
Woody vegetation	−0.20	−0.50	0.07	−0.54	−1.41	0.09	**−2.74**	**−5.43**	**−0.70**	−0.11	−0.54	0.28
Woody vegetation^2^	**−0.33**	**−0.61**	**−0.09**	**−0.67**	**−1.46**	**−0.10**	**−1.96**	**−3.61**	**−0.62**	−0.01	−0.42	0.36
Bare ground	**0.81**	**0.62**	**1.01**	0.09	−0.27	0.44	**1.26**	**0.82**	**1.78**	**1.03**	**0.63**	**1.50**
Bare ground^2^	−0.18	−0.36	0.00	−0.05	−0.36	0.27	−0.27	−0.67	0.20	−0.22	−0.56	0.18
Stones	**1.15**	**0.96**	**1.37**	**0.41**	**0.10**	**0.73**	**1.58**	**1.10**	**2.18**	**1.80**	**1.31**	**2.35**
Stones^2^	**−0.49**	**−0.67**	**−0.29**	**−0.45**	**−0.73**	**−0.17**	−0.37	−0.79	0.13	**−0.95**	**−1.34**	**−0.52**
Snow				**−2.92**	**−4.54**	**−1.72**						
Snow^2^				**−1.18**	**−1.84**	**−0.66**						
Vegetation height	**−1.04**	**−1.29**	**−0.80**	**−1.10**	**−1.58**	**−0.65**	**−1.59**	**−2.18**	**−1.05**	**−1.21**	**−1.69**	**−0.78**
Distance to marmot burrow	**−0.42**	**−0.64**	**−0.21**	**−0.37**	**−0.71**	**−0.05**	−0.44	−0.92	0.01	**−0.68**	**−1.18**	**−0.20**
NDVI	**0.49**	**0.29**	**0.70**	0.08	−0.31	0.47	**0.71**	**0.31**	**1.16**	**0.50**	**0.07**	**0.96**
NDVI^2^	0.01	−0.16	0.18	0.03	−0.30	0.36	0.26	−0.11	0.68	0.02	−0.34	0.36
Rate of NDVI change	**−0.29**	**−0.49**	**−0.10**	−0.18	−0.49	0.13	**−0.62**	**−1.10**	**−0.18**	**−0.40**	**−0.82**	**−0.01**
Rate of NDVI change^2^	0.03	−0.14	0.19	−0.18	−0.51	0.14	−0.30	−0.72	0.09	0.27	−0.13	0.66

*Note*: Foraging versus pseudo‐absence points were modeled using a logistic regression with logit‐link function. Bird ID and point ID are random effects, while all other variables are fixed effects. Given are the estimate and lower (2.5%) and upper (97.5%) limits of the 95% credible interval. Estimates where the 95% credible interval does not contain zero are highlighted in bold.

**FIGURE 3:**
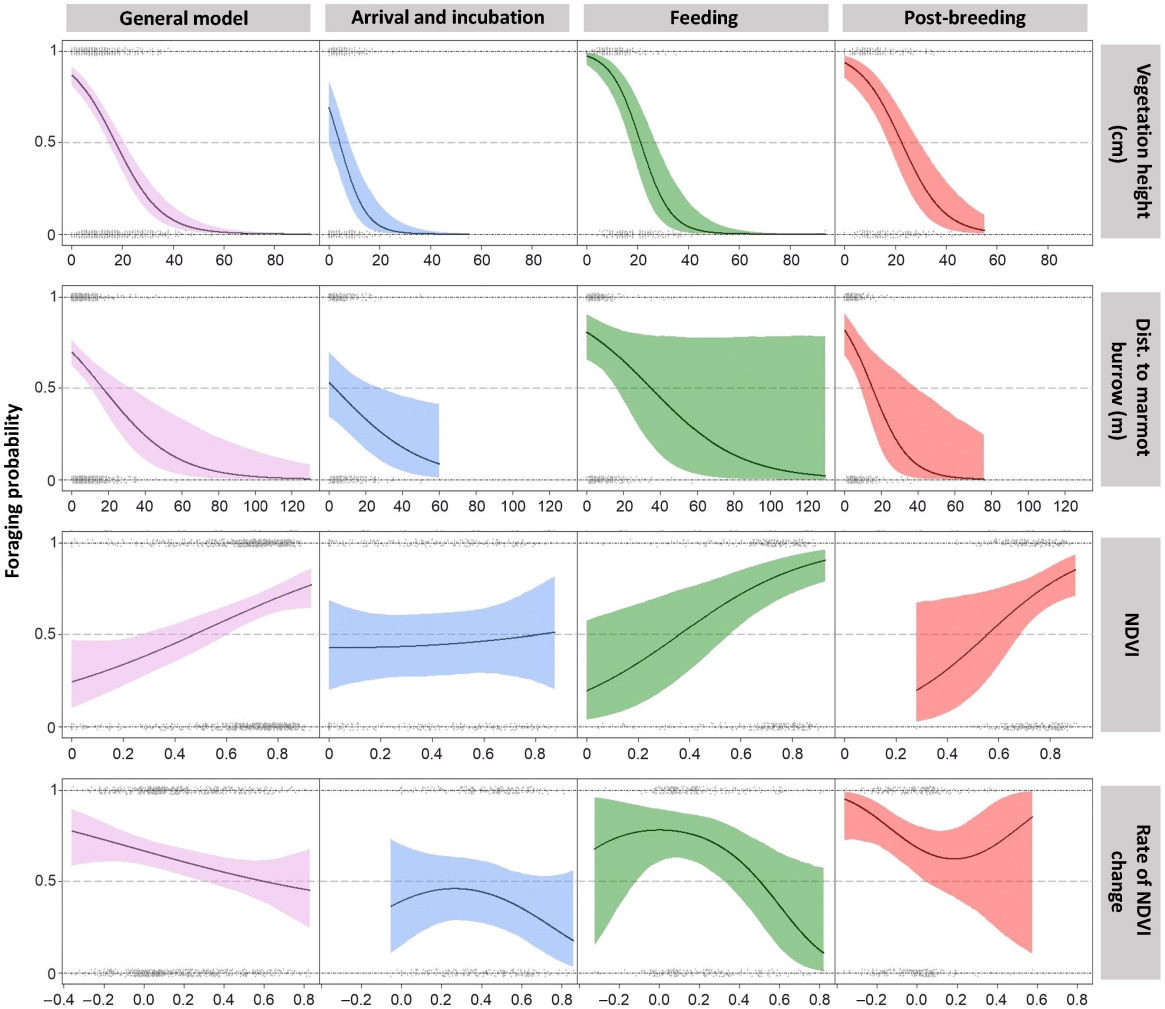
Model predictions from logistic regression models showing the average effect (solid line) of vegetation height, distance to marmot burrow, NDVI, and the rate of NDVI change (labeled on the righthand side of the plots) on the foraging probability (presence vs. pseudo‐absence) for the general model (whole study period; first column) and each period separately (other columns) within 1 m of the foraging (presence = 1) and pseudo‐absence (0) locations. The colored areas represent the 95% Bayesian credible intervals and the gray dots show the raw data.

Increasing distance to the closest marmot burrow had a negative effect throughout the study period (Table 2, Figure 3), indicating a preference for foraging locations close to burrows (Figure 3). This effect was strongest at the postbreeding stage (Table 2). Except for the arrival and incubation stage, NDVI had a positive linear effect in each model, being strongest during the feeding period (Table 2, Figure 3). Even though vegetation was greening and growing fast during arrival and incubation (Figure S5), the rate of NDVI change had no strong effect on the foraging probability at that stage (Table 2), but it had a negative effect in the other models (Table 2, Figure 3). In the postbreeding stage, the rate of NDVI change had a slightly negative linear effect (Table 2, Figure 3).

We ran all models based on a 2‐m‐radius with very similar results (Table S2, Figure S6): Although some effects were stronger on the smaller scale, the general patterns were the same (Table 2, Table S2, Figure S6).

## 4 DISCUSSION

Our study highlights the importance of small‐scale characteristics in the foraging preferences of a long‐distance migrant breeding in high‐alpine habitats. Accessibility to the ground and habitat heterogeneity determined, on a very fine scale, whether a location was chosen for foraging. Habitat structure and ground cover composition changed as the season advanced, but northern wheatears generally showed similar habitat preferences throughout their presence in the study area. Interestingly, the habitat preferences were consistent between females and males as well as between adults and juveniles. We found a specific preference for open patches, interspersed with stones within vegetated areas, where prey abundance is expected to be higher (Morris, 2000). This underpins that a diverse habitat is necessary to sustain food availability for northern wheatears throughout their stay on the Alpine breeding grounds. Preferred foraging habitat in the study area was composed of multiple types of ground cover. Especially the presence of bare ground patches seemed important, which allow birds to detect and access prey more easily than in the surrounding vegetation (Schaub et al., 2010; Vickery & Arlettaz, 2012). In particular, bare ground plays a crucial role during food provisioning for chicks, when food demand is enhanced and vegetation is growing fast (Moreno, 1989b). Rocks and boulders may have played a similar role, as they served as perching positions, allowing the birds to detect prey more easily. Particularly in the postbreeding period, stones may also have hosted an increased amount of prey, as we have repeatedly observed birds picking ants and other prey items from boulders or directly from anthills located in rocky areas; this was not the case earlier in the season.

Nonetheless, our NDVI results indicate that vegetation productivity is an important component of the foraging microhabitat. This result must be interpreted in the context of larger‐scale effects. The minimal spatial resolution of sentinel‐2 satellite data is 10 m, which means that the available information summarizes a larger area than the sampling locations, informing on the productivity in the habitat matrix around the foraging location. Even though patches with bare ground and stones were preferred on a small scale, they lay within the territories in the study area where heterogeneous and productive grassland is the dominating habitat type. On the one hand, this result implies that northern wheatears selected productive areas for foraging that offer high arthropod abundance and diversity (Morris, 2000), which increases with vegetation height (Atkinson et al., 2004). On the other hand, high vegetation decreases visibility and access to the ground (Atkinson et al., 2004; Vickery & Arlettaz, 2012) and reduces the probability of a foraging attempt being successful (Dennis et al., 2008). As a ground‐foraging insectivore, the northern wheatear requires visibility of and access to the ground for foraging (Arlt & Pärt, 2007; van Oosten et al., 2014). The preference for short vegetation on a fine scale suggests that prey accessibility is more limiting for successful foraging than prey abundance. This result is consistent with findings from study sites in the lowland of Northern Europe. In the Netherlands, where prey abundance remains stable throughout the breeding season, northern wheatears are more limited by prey accessibility than by prey abundance, as they preferentially forage in short grass (van Oosten et al., 2014). Similarly, northern wheatear populations had improved growth rates in short‐vegetation habitats compared to tall field layers in Swedish farmland (Arlt et al., 2008), where the presence of short vegetation is a major driver of population growth (Arlt et al., 2008; Paquet et al., 2019) and an important clue for habitat selection (Arlt & Pärt, 2007). The preference for short vegetation has been consistently described for lowland bird communities (Atkinson et al., 2004; Rime et al., 2020; Vickery & Arlettaz, 2012) as well as for other insectivorous alpine birds (Barras et al., 2020; Brambilla et al., 2017; Resano‐Mayor et al., 2019).

Even though woody vegetation reduces ground accessibility and was usually avoided, it played a specific role later in the season when it provided berries as an additional food source, explaining the observed weaker avoidance of this habitat type in the postbreeding season. We then observed northern wheatears foraging on *Vaccinium myrtillus* and *Daphne mezereum* berries. Coloring of the feces confirmed the consumption of berries (García‐Rodríguez et al., 2022). Many insectivorous birds become frugivorous when their main food source becomes scarce (Bairlein, 2003; Fry, 1992). Berries are important sources of nutrients that may enhance molt and are crucial for migration (Bairlein, 2003; Eeva et al., 2018). Berries are therefore actively chosen, while including berries in an insectivorous diet most likely also reduces foraging energy expenditure and further supports fattening for migration (Lindström, 2003). Nevertheless, northern wheatears still preferred open habitat in the postbreeding period, suggesting a sufficient abundance of arthropods (Beck et al., 2010; Pilar et al., 2020; Resano‐Mayor et al., 2019).

The preference for highly accessible patches within more productive areas has been described for a variety of ground‐foraging insectivorous farmland birds (Atkinson et al., 2004; Martinez et al., 2010; Schaub et al., 2010; Tagmann‐Ioset et al., 2012; Vickery et al., 1999; Vickery & Arlettaz, 2012; Weisshaupt et al., 2011) as well as for alpine specialists (Barras et al., 2020; Brambilla et al., 2017; Resano‐Mayor et al., 2019). Food abundance for insectivores is higher in heterogeneous habitat (Cole et al., 2010), and fine‐scale habitat diversity provides accessible patches within species‐rich landscapes that support high food abundance (Atkinson et al., 2004; Vickery & Arlettaz, 2012). Furthermore, habitat heterogeneity maintains food availability temporally by allowing diverse vegetation phenology to coexist and supply sufficient food throughout the season (Benton et al., 2003; Hovick et al., 2015; Vickery & Arlettaz, 2012). This is important because the habitat characteristics in the study area were strongly influenced by seasonal changes, while the species' foraging habitat preferences remained similar. The availability of suitable foraging habitat mainly depended on the progress of spring greening‐up. In 2021, the area experienced a late and cold spring. When the birds arrived in the breeding region in May, most of their territories were still covered by snow. During the melting period, the edges of snow fields played an important role, providing accessible habitat with high prey abundance (Barras et al., 2020; Brambilla et al., 2017; Leingärtner et al., 2014; Resano‐Mayor et al., 2019). Once vegetation growth increased and ground accessibility declined, habitat heterogeneity and the availability of open habitat patches became crucial in providing suitable foraging habitat. Similar results were found at a larger scale in Swedish farmland habitats, where fields with low vegetation became increasingly important for northern wheatears and positively influenced reproductive success later in the season (Arlt & Pärt, 2007). Sander et al. (2022) showed that nest survival of northern wheatears benefitted from a higher vegetation at another site in the Alps with a broader elevational gradient. This could be explained by a sparser and generally lower vegetation in more mineral‐based high‐elevation habitats. This is in line with the preference for more productive grasslands with an intermediate ground cover of stones and rock in our study area. At our study site, most northern wheatears remained in their territories throughout their presence (Rime et al., 2023). Other ground‐dwelling insectivorous birds seem to be less capable of finding suitable habitat in their breeding territories as the season advances. For example, white‐winged snowfinches (*Montifringilla nivalis*) rely on Tipulidae larvae at the retreating snow front (Brambilla et al., 2017; Resano‐Mayor et al., 2019), a food resource used by adult northern wheatears only in the pre‐breeding period, while ring ouzels (*Turdus torquatus alpestris*) rely mainly on earthworms and perform diel and seasonal altitudinal movements to track suitable foraging habitat as spring advances (Barras et al., 2020, 2021). Similarly, water pipits (*Anthus spinoletta*) perform within‐season movements to avoid dense and high grassland as vegetation growth progresses (Ceresa et al., 2020).

Due to this strong dependence of northern wheatears on the small‐scale habitat mosaic that maintains suitable foraging habitat, the species is likely sensitive to climate and land‐use change (Scridel et al., 2018; Theurillat & Guisan, 2001). In most parts of Europe, northern wheatear populations are declining, while the Alpine populations are stable overall (Gideon et al., 2014; Hallman et al., 2022; Issa & Muller, 2015; Keller et al., 2020; Knaus et al., 2018). Northern wheatears might be less vulnerable to climate change than other high‐elevation specialists as long as micro‐habitat heterogeneity is maintained. The rock ptarmigan (*Lagopus muta*) and the white‐winged snowfinch (*Montifringilla nivalis*) for instance show a decrease in all but the uppermost part of their distributional range where populations remain stable (Issa & Muller, 2015; Keller et al., 2020; Knaus et al., 2018). The population trends suggest that these species are limited in their ability to find suitable habitat, even at higher altitudes.

For northern wheatears, an upward shift in elevation has been observed in Switzerland, with the increase above 2400 m being higher than the loss at lower elevation, resulting in a stable or increasing general population trend (Hallman et al., 2022; Knaus et al., 2018). However, such an ongoing shift could lead to range contraction in the future (Dirnböck et al., 2003; Jähnig et al., 2020). Furthermore, winters in the Alps tend to become shorter, and spring greening‐up is expected to advance earlier (Asam et al., 2018; Chamberlain & Pearce‐Higgins, 2013; Gobiet et al., 2014). The resulting rise of the tree line, the increasing bush encroachment, and higher vegetation density are threatening the availability of accessible foraging habitat also for the northern wheatear (Ceresa et al., 2021; Jähnig et al., 2020). Land‐use change enhances population threats even further (Kulakowski et al., 2011): Agricultural intensification leads to landscape homogenization (Benton et al., 2003) and to higher nutrient levels, accelerating vegetation development and altering species composition (Dirnböck et al., 2003; Fischer et al., 2008). Even more apparent in alpine regions, pastoral abandonment leads to an increased vegetation height and eventually to shrub and forest encroachment (Gehrig‐Fasel et al., 2007; Kulakowski et al., 2011; Laiolo et al., 2004). On the other hand, low‐intensity grazing of cattle positively influences ground‐foraging birds (Atkinson et al., 2004; Laiolo et al., 2004; Vickery et al., 1999) and maintains suitable foraging habitat for the northern wheatear (Maron & Lill, 2005). It is important to note that the positive effects of grazing on grassland bird communities are associated with low‐intensity grazing, as applied in our study area, whereas high‐intensity grazing can negatively affect them (Brambilla et al., 2020; Garcia‐Pausas et al., 2017). Even though we were not able to quantify the effects of grazing with our method focusing on small‐scale habitat parameters, low‐intensity grazing is an important driver of landscape dynamics (Laiolo et al., 2004; Yoshihara et al., 2010). In areas that were grazed, vegetation height was lower and more heterogenous, and the growing dynamic was disrupted. With the onset of grazing in the study area, mean vegetation height stopped increasing and leveled off. Additionally, northern wheatears showed a preference for patches with stable vegetation dynamics that ensure long‐term habitat heterogeneity (Hovick et al., 2015; Vickery & Arlettaz, 2012). Furthermore, the foraging habitat of northern wheatears was probably positively influenced by alpine marmots, as northern wheatears where often foraging close to their burrows. Despite field observations suggesting marmots as potential nest predators, benefits of association with marmots seem to persist. Marmots maintain structural heterogeneity and accessible habitat by creating patches of bare ground, keeping the vegetation short, and potentially improving arthropod abundance and species richness (Ballová et al., 2019; Buyandelger et al., 2021; Buyandelger & Otgonbayar, 2022; Davidson et al., 2012).

Even though resource availability and habitat characteristics change temporally within a season, the foraging habitat preferences of northern wheatears remained similar at the study site. Northern wheatears depend on the availability of suitable foraging habitat within the same territory for the entire presence at the breeding site, even after the chicks are fully independent. Within an ecosystem that is characterized by spatiotemporal dynamics that are different to those in lowland habitats, Alpine northern wheatears inhabit an ecological niche that features a mosaic of accessible patches within vegetated areas that provide high prey abundance. Due to pressures from climate and land‐use change on alpine ecosystems, this habitat is fragile and northern wheatears may be sensitive to habitat loss and range contraction. Our study emphasizes the importance of the Alpine breeding area for northern wheatears. It underlines the necessity to maintain and preserve the spatiotemporal availability of structural diversity and small‐scale habitat heterogeneity that is critical in providing suitable foraging habitat for northern wheatears in the Alps in the long term.

## AUTHOR CONTRIBUTIONS

**Christoph M. Meier:** Conceptualization (supporting); formal analysis (supporting); funding acquisition (equal); methodology (equal); project administration (equal); resources (equal); supervision (equal); writing – original draft (supporting); writing – review and editing (equal). **Florian Knaus:** Conceptualization (equal); formal analysis (supporting); funding acquisition (equal); methodology (equal); project administration (equal); resources (equal); supervision (equal); writing – original draft (supporting); writing – review and editing (equal). **Pius Korner:** Data curation (supporting); formal analysis (equal); methodology (supporting); software (equal); visualization (supporting); writing – original draft (supporting); writing – review and editing (equal). **Barbara Helm:** Funding acquisition (equal); project administration (supporting); resources (supporting); supervision (supporting); writing – original draft (supporting); writing – review and editing (equal). **Valentin Amrhein:** Supervision (supporting); validation (supporting); writing – review and editing (equal). **Yann Rime:** Conceptualization (lead); data curation (supporting); formal analysis (equal); funding acquisition (lead); investigation (supporting); methodology (lead); project administration (lead); resources (equal); software (equal); supervision (lead); validation (equal); visualization (equal); writing – original draft (supporting); writing – review and editing (equal). **Thomas M. Müller:** Conceptualization (supporting); data curation (lead); formal analysis (lead); investigation (lead); methodology (equal); project administration (equal); software (equal); validation (equal); visualization (lead); writing – original draft (lead); writing – review and editing (lead).

## FUNDING INFORMATION

This study was funded by the Swiss Ornithological Institute (Vogelwarte Sempach) and the Swiss Federal Institute of Technology Zurich (ETH Zurich).

## CONFLICT OF INTEREST STATEMENT

The authors declare that they have no conflict of interest.

## Supporting information


Appendix S1.

## Data Availability

The data and codes used in this study are deposited on Zenodo under the DOI: 10.5281 at https://doi.org/10.5281/zenodo.7805040 (Müller et al., 2023).
