# Supplementary material for: Finding food in a changing world: Small‐scale foraging habitat preferences of an insectivorous passerine in the Alps
Source: Ecol Evol. 2023 May 17;13(5):e10084. doi: 10.1002/ece3.10084 (PMC10191804; doi:10.1002/ece3.10084)
Supplement: Supplementary file 1 — Appendix S1. [file ECE3-13-e10084-s001.pdf]

Supplementary Material

Table S1: Variables recorded or calculated for each point but not included in the multivariate statistical models.

Category	Variable	Description	Unit
Point information	Coordinates	X and Y coordinates of the point location	X, Y
Bird Information	Age	Age class (juvenile, adult)	
	Sex	Adult sex (female, male)	
	Stage	Breeding stage (arrival, incubation, feeding, post-breeding)	
Nest	Nest ID	Unique identifier for the individual's nest	
	Distance to Nest	3D-distance from point to nest	m
Ground cover	Live vegetation	Green vegetation grown this year	%
Snow	Distance to snow	Distance to closest snow patch (if within 100 m)	m
Ecosystem Engineers	Cows	Cows present within 5m radius (Yes/No)	2 categories
	Grazing	Meadows ungrazed, currently grazed, previously grazed this year or grazed in the previous years	4 categories
	Cow dung	Presence of cow dung within the radius	2 categories
Topography	Elevation	Altitude	m. a. s. l.
	Slope	Slope inclination (steepness)	%
	Aspect	Orientation	°
Berries	Number of berries	Estimated number of berries within the radius	Number of berries

Table S2: Summary of the output of the **general** (all-season) model, **arrival and incubation**, **feeding** and **post-breeding** model using the **2-m** data. Foraging vs. pseudo-absence points were modelled using a logistic regression with logit-link-function. Bird ID and point ID are random effects, while all other variables are fixed effects. Given are the estimate and lower (2.5%) and upper (97.5%) limits of the 95% Credible Interval. Estimates where the 95% Credible Interval does not contain zero are highlighted in bold.

	General model			Arrival and incubation			Feeding			Post-breeding		
Variable	Estimate	CrI 2.5%	CrI 97.5%	Estimate	CrI 2.5%	CrI 97.5%	Estimate	CrI 2.5%	CrI 97.5%	Estimate	CrI 2.5%	CrI 97.5%
bird ID (Intercept)	0.11	0.00	0.32	**0.16**	**0.01**	**0.46**	**0.24**	**0.01**	**0.69**	**0.19**	**0.01**	**0.55**
point ID (Intercept)	0.08	0.00	0.23	0.13	0.00	0.37	**0.18**	**0.01**	**0.51**	**0.20**	**0.01**	**0.57**
dead vegetation	-0.14	-0.35	0.05	-0.30	-0.67	0.07	0.05	-0.28	0.36	**-0.51**	**-0.90**	**-0.14**
dead vegetation^2	0.08	-0.11	0.25	-0.09	-0.40	0.20	0.12	-0.20	0.41	-0.13	-0.57	0.26
woody vegetation	0.06	-0.14	0.25	-0.08	-0.45	0.24	-0.30	-1.05	0.28	0.03	-0.33	0.36
woody vegetation^2	-0.20	-0.41	-0.00	-0.21	-0.54	0.08	-0.55	-1.30	0.03	-0.08	-0.43	0.25
bare ground	0.71	0.52	0.90	0.01	-0.31	0.35	**1.18**	**0.76**	**1.64**	**0.75**	**0.41**	**1.13**
bare ground^2	-0.16	-0.32	0.02	-0.05	-0.34	0.24	-0.13	-0.51	0.35	-0.26	-0.56	0.07
stones	**1.07**	**0.87**	**1.30**	**0.30**	**0.03**	**0.58**	**1.26**	**0.90**	**1.68**	**1.62**	**1.18**	**2.12**
stones^2	**-0.35**	**-0.53**	**-0.17**	**-0.35**	**-0.60**	**-0.09**	-0.33	-0.68	0.06	**-0.61**	**-1.00**	**-0.16**
snow				**-2.04**	**-3.06**	**-1.18**						
snow^2				**-0.89**	**-1.45**	**-0.43**						
vegetation height	**-1.02**	**-1.26**	**-0.79**	**-1.00**	**-1.41**	**-0.58**	**-1.36**	**-1.84**	**-0.92**	**-1.23**	**-1.67**	**-0.80**
distance to marmot burrow	**-0.44**	**-0.68**	**-0.22**	**-0.34**	**-0.64**	**-0.04**	**-0.48**	**-0.93**	**-0.05**	**-0.67**	**-1.15**	**-0.22**
NDVI	**0.39**	**0.19**	**0.60**	-0.01	-0.37	0.37	**0.56**	**0.18**	**0.93**	**0.57**	**0.12**	**0.99**
NDVI^2	0.06	-0.11	0.23	0.06	-0.26	0.38	**0.34**	**0.01**	**0.69**	0.09	-0.26	0.42
rate of NDVI change	**-0.32**	**-0.51**	**-0.13**	-0.24	-0.54	0.04	**-0.66**	**-1.06**	**-0.27**	**-0.41**	**-0.81**	**-0.02**
rate of NDVI change^2	-0.05	-0.22	0.11	-0.17	-0.46	0.13	-0.20	-0.55	0.14	0.25	-0.15	0.64

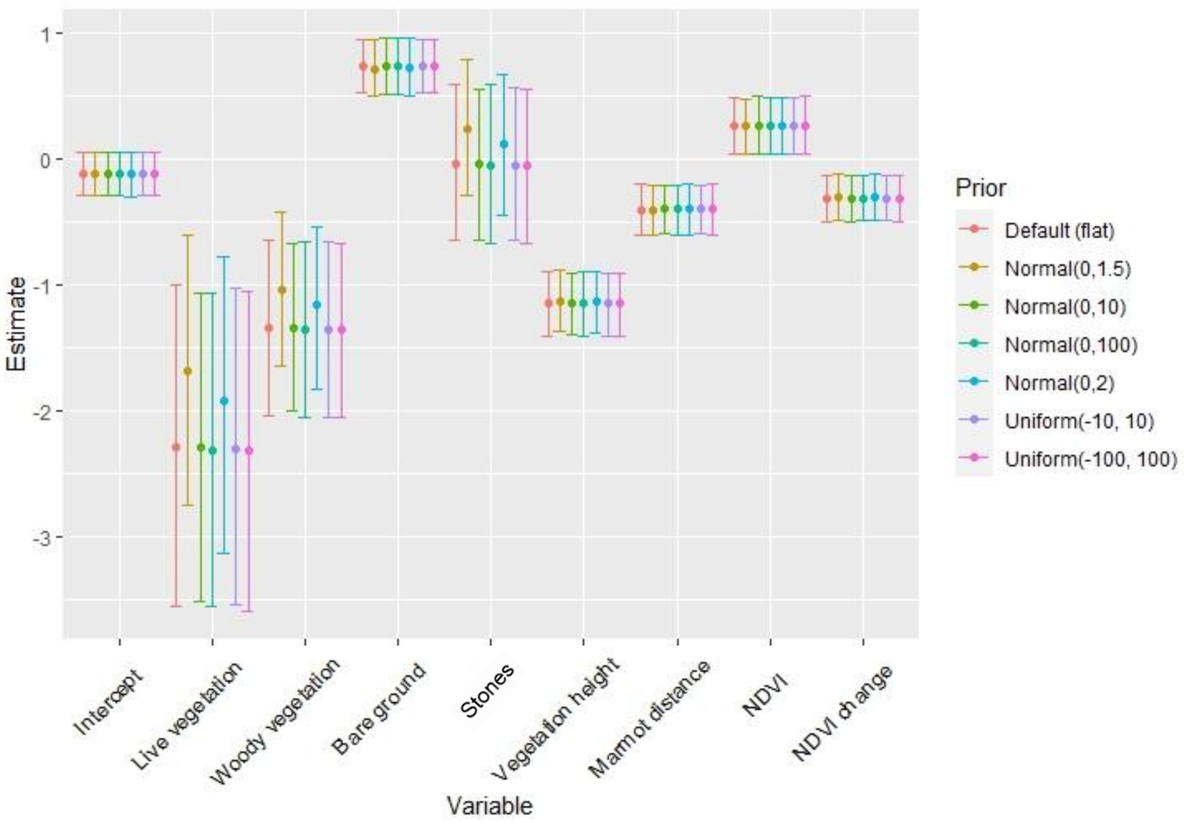

Fig. S1: Sensitivity analysis plot showing the estimates and 95%-credible intervals (CrI) for different prior distributions for the population-level effects of a preliminary model. The two very informative priors N(0,1.5) and N(0,2) influence the results quite strongly while the uninformative priors lead to very similar results. As a conclusion, we chose flat priors (N(0,100)).

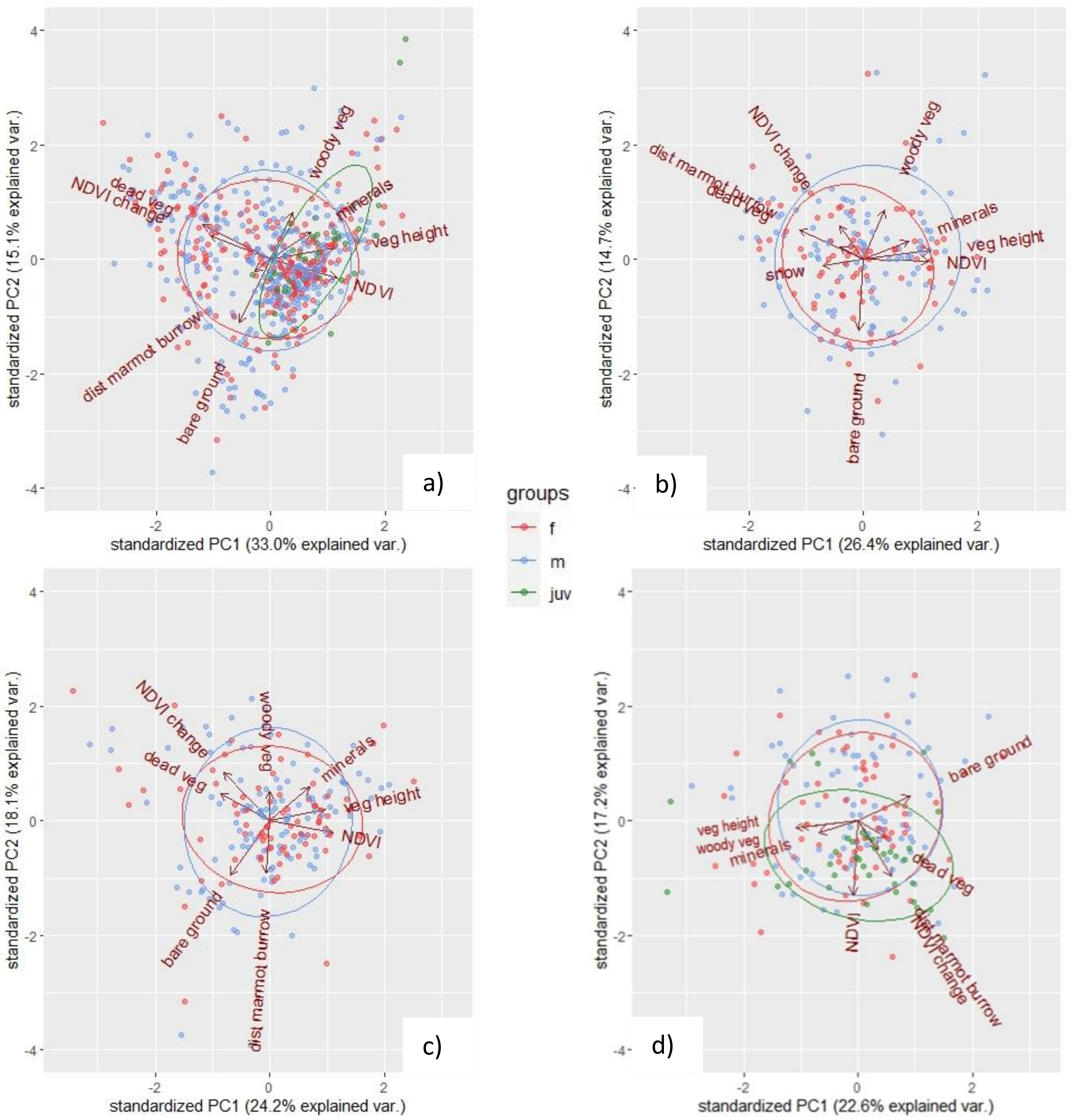

Fig. S2: Principal Component Analysis showing the first 2 principal components (PC1 & PC2) with foraging data (presence points only) of the entire study period (general model) (a), arrival and incubation (b), feeding (c) and post-breeding periods (d) and the variables used in the respective models. Colours distinguish points of females (red), males (blue) and juveniles (green), and the coloured circles group the respective categories together. Juveniles were only present in the post-breeding period and therefore do not occur in the arrival & incubation and the feeding PCA. The variation within the model stage that is explained by each of the principal components is given on the x (PC1) and y (PC2) axis.

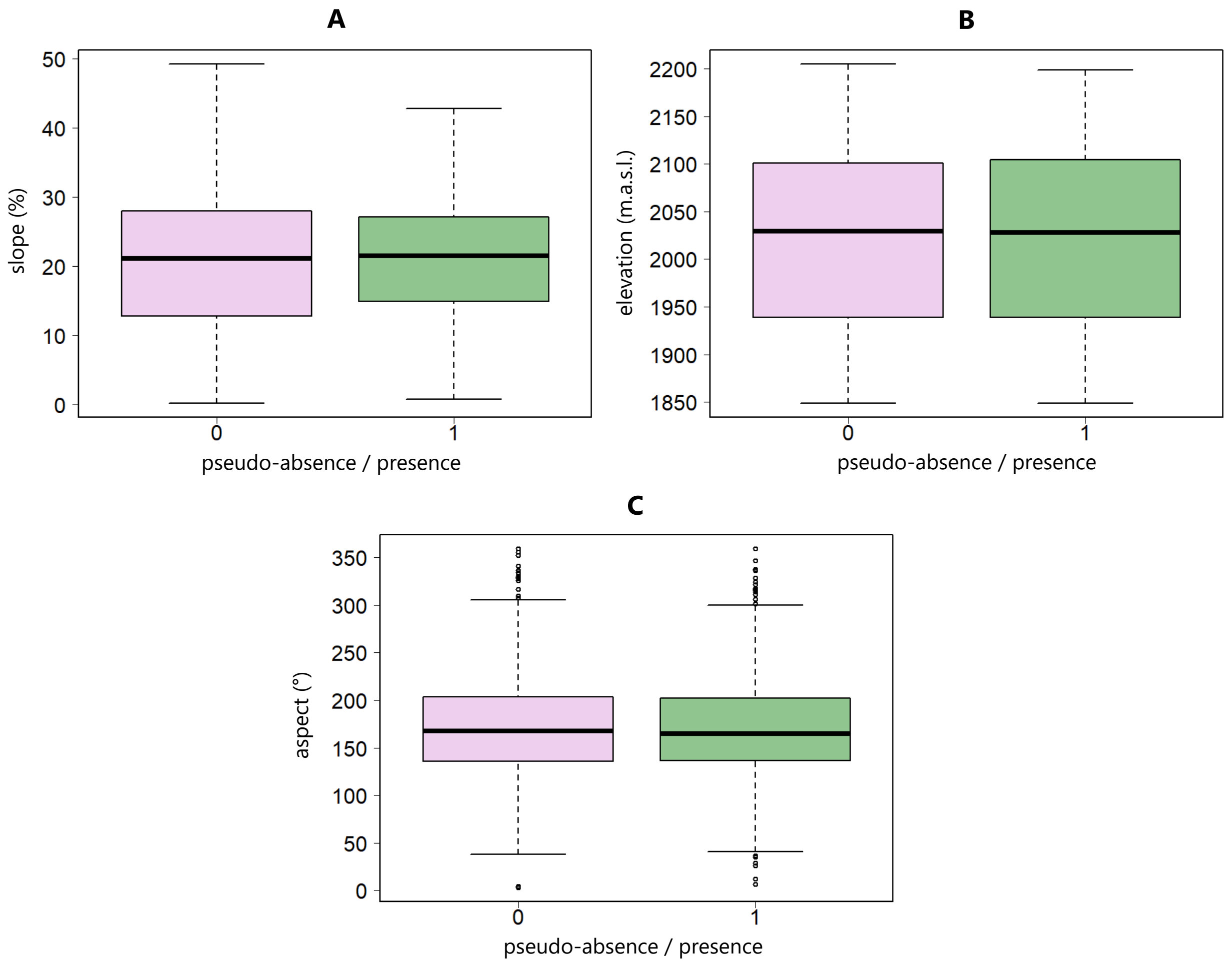

Fig. S3: Boxplots showing the distribution of the topgographic variables for presence (1) and pseudo-absence (0) points from the entire study season. Plot A shows the slope (%), B shows the elevation (m.a.s.l.) and C shows the aspect (°). The bold black line indicates the median and the boxes show the interquartile ranges. The dotted line covers the range of datapoints. Univariate mixed models (using Bird ID and Point ID as random factors) for topographic variables were additionally run. They revealed no strong effects of the slope (Estimate: 0.06, 95%-CrI: -0.06 to 0.18), elevation (Estimate: 0.01; 95%-CrI: -0.11 to 0.13), and aspect (Estimate: -0.02; 95%-CrI: -0.14 to 0.1) on the foraging probability.

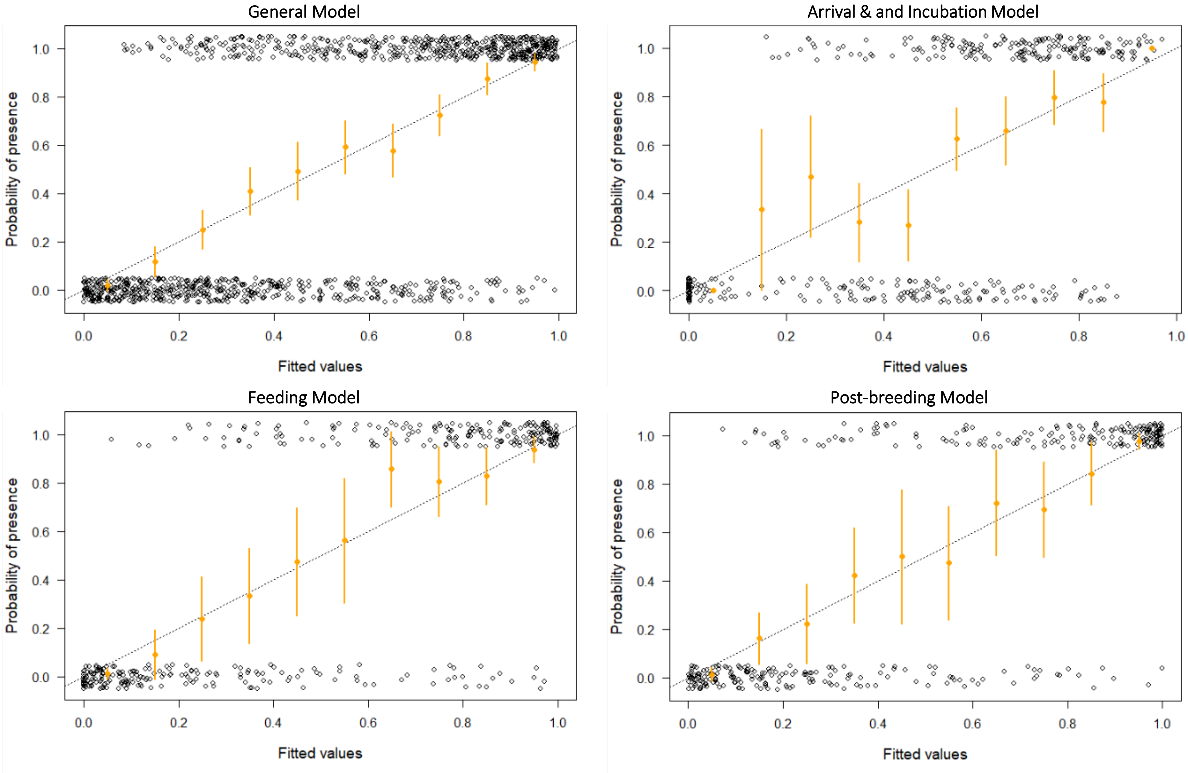

Fig. S4: Goodness of fit plots showing how well the fitted values match the data in each model. The open circles show observed foraging (presence=1) and pseudo-absence (absence=0) points. Orange dots indicate the mean (and 95% confidence intervals as orange lines) of observations on an interval of 0.1. Perfect coincidence between observed and fitted values is indicated by the dotted line.

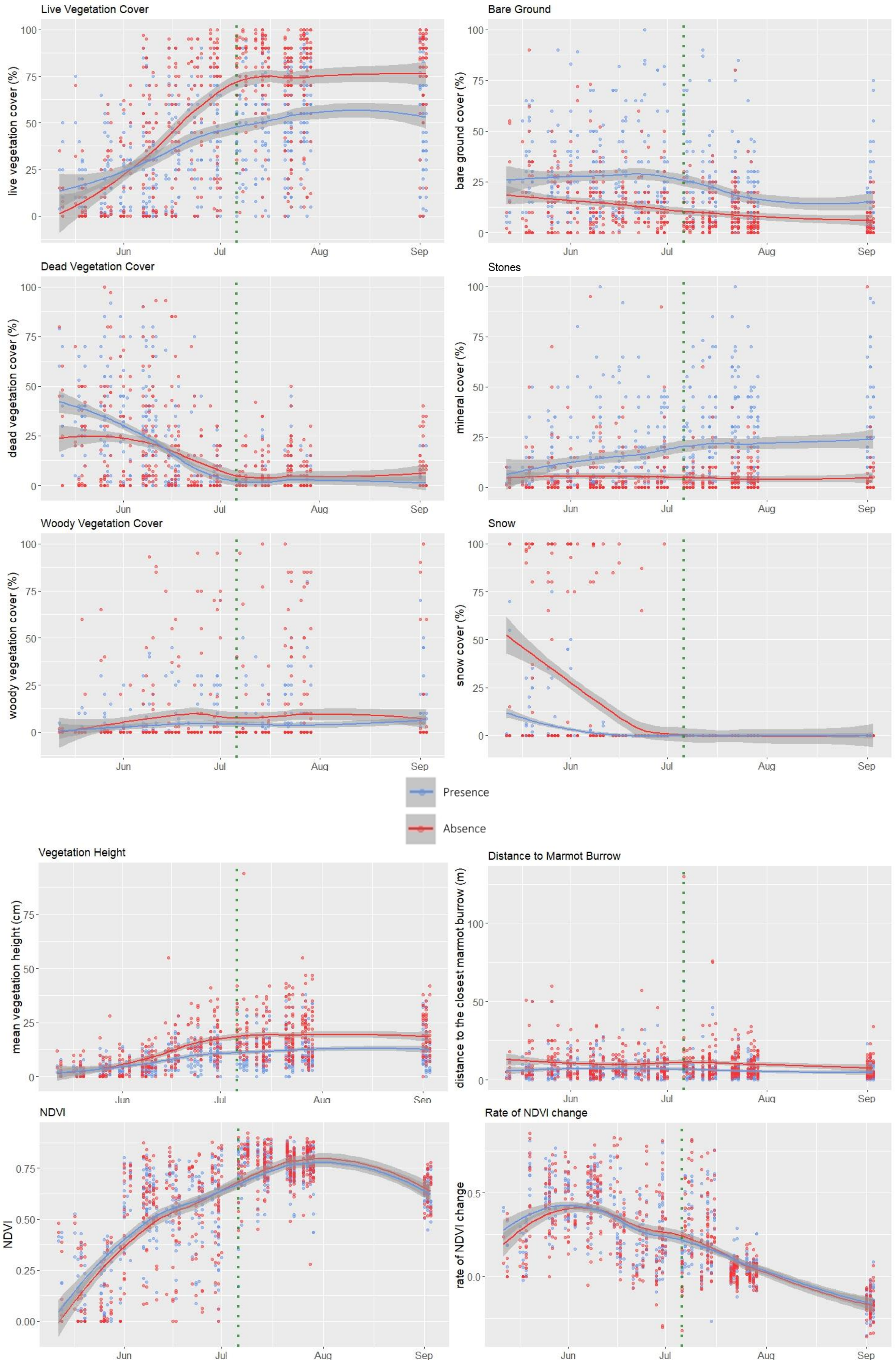

Fig. S5: Seasonal development of each variable on the 1-m radius. The coloured points represent the foraging (blue) and pseudo-absence points (red). The solid lines show the mean development at foraging (blue) and pseudo-absence points (red) with the standard deviation (grey area). The green dotted line indicates the onset of grazing in the study area.

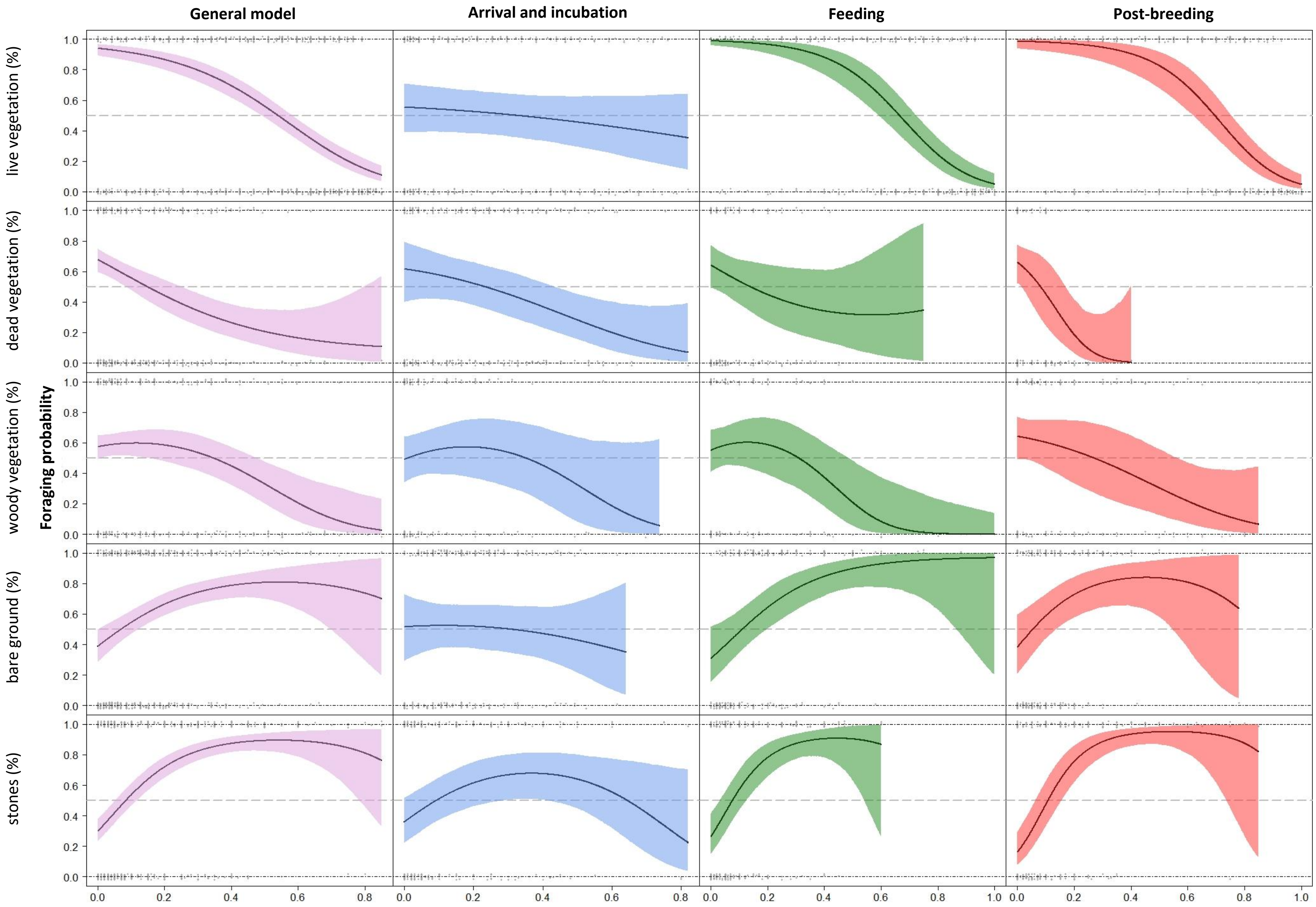

Fig. S6: Model predictions from logistic regression models showing the average effect (solid line) of each variable on the foraging probability (presence vs. pseudo-absence) for the general model (whole study period; 1st column) and each period separately (2nd-4th column) using the 2-m data. "Live vegetation" was not used as a predictor in the model but it is a derived parameter from the other ground cover parameters and given because all ground covers add up to 1. The coloured areas represent the 95% Bayesian Credible Intervals and the grey dots show the raw data. The plot is continued on the next page.

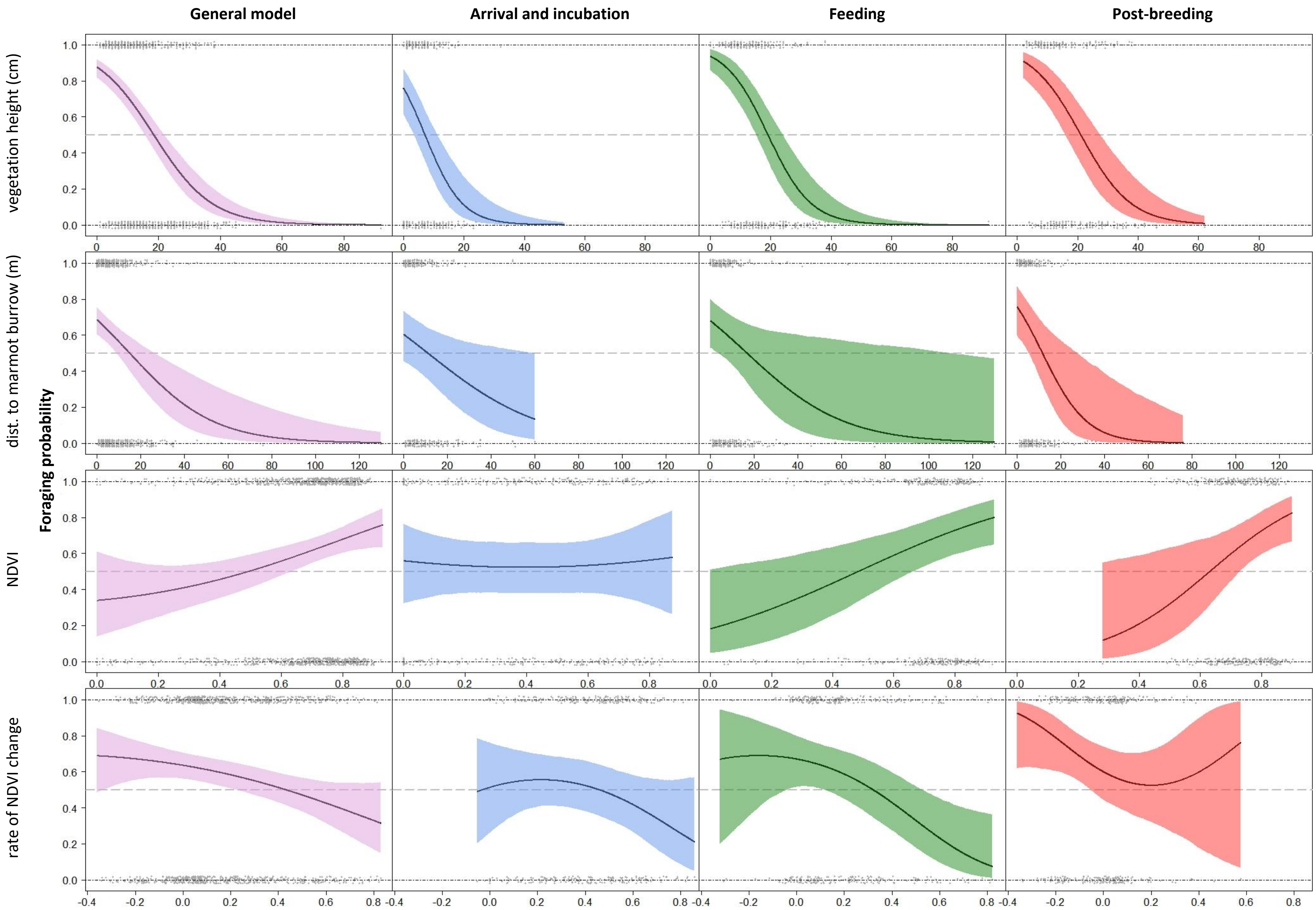

General model
Arrival and incubation
Feeding
Post-breeding
Foraging probability
vegetation height (cm)
dist. to marmot burrow (m)
NDVI
rate of NDVI change
